# Metabolic Syndrome, Hormone Levels, and Inflammation in Patients with Erectile Dysfunction

**DOI:** 10.1100/2012/272769

**Published:** 2012-09-02

**Authors:** Miguel Ángel Arrabal-Polo, Salvador Arias-Santiago, Fernando López-Carmona Pintado, Sergio Merino-Salas, Clara Lahoz-García, Armando Zuluaga-Gómez, Miguel Arrabal-Martin

**Affiliations:** ^1^Urology Department, San Cecilio University Hospital, Camino de Ronda street, 143, 18003 Granada, Spain; ^2^Department of Medicine, Faculty of Medicine, Granada University, Granada, Spain

## Abstract

*Background.* The end point of this study was to investigate the prevalence of MS in patients with ED in comparison with control subjects and to analyse the association with acute phase reactants (CRP, ESR) and hormone levels. *Methods.* This case-control study included 65 patients, 37 with erectile dysfunction, according to the International Index of Erectile Function (IIEF) from the Urology Department of San Cecilio University Hospital, Granada (Spain) and 28 healthy controls. The prevalence of metabolic syndrome was calculated according to ATP-III criteria. Hormone levels and acute phase parameters were studied in samples drawn. *Results.* The ATP-III criteria for MS were met by 64.9% of the patients with ED and only 9.5% of the controls (*P* < 0.0001, OR = 17.53, 95% CI: 3.52–87.37). Binary logistic regression analysis showed a strong association between patients with ED and MS, even after additional adjustment for confounding factors (OR = 20.05, 95% CI: 1.24–32.82, *P* < 0.034). Patients with hypogonadism presented a significantly higher prevalence of metabolic syndrome. Multiple linear regression analysis showed that systolic BP and CRP predicted 0.46 (model *R* ^2^) of IIEF changes. *Conclusion.* Chronic inflammation found in patients with ED might explain the association between ED and metabolic syndrome.

## 1. Introduction

Erectile dysfunction (ED) is defined as the inability to maintain an adequate erection for satisfactory sexual intercourse [1]. The degree of erectile dysfunction can be objectified by the international index of erectile function (IIEF), which allows us to classify ED as mild, moderate, or severe based on the score [2]. Related causes of erectile dysfunction are variable and can include vascular, endocrinological, neurological, and psychological causes [3]. The most common causes often involve alterations in the vascular endothelium due to atherosclerosis, which is a common physiopathological link between ED and cardiovascular disease (CD). Endothelial damage results in the reduced formation of nitric oxide, thereby decreasing blood flow, and negatively impacting erectile function [4]. Metabolic syndrome (MS) is associated with endothelial dysfunction and is defined by a number of components such as high blood pressure, elevated triglycerides, low HDL-cholesterol, increased abdominal circumference, or insulin resistance manifested as diabetes mellitus or glucose intolerance [5]. Metabolic syndrome is important because it may confer an overall cardiovascular risk that is higher than the individual components; subjects who met ATP-III MS criteria had a 2.59-fold greater likelihood (OR = 2.59) of experiencing a cardiovascular event in the next 10 years [6]. The severity of ED has been associated with a higher occurrence of major cardiovascular events and an increased risk of serious cardiovascular events [7]. Some inflammatory mediators, like C-reactive protein and fibrinogen, are elevated in patients with CD, especially in those with coronary heart disease. These mediators have clinical significance and could be useful in monitoring the treatment of these patients [8], but additional studies are necessary to confirm these results. Chronic inflammation plays an important role in the development of insulin resistance, endothelial dysfunction, and cardiovascular disease. Other studies report that plasma acute-phase protein levels are elevated in patients with ED (fibrinogen, von Willebrand factor, and interleukins) [9]. 

Recent studies have revealed that testosterone plays a protective role in the development of endothelial damage, and a negative linear relationship between testosterone levels and the severity of coronary disease has been found [10, 11]. Testosterone also plays an important role in erectile function and sexual desire, but it is not necessarily decreased in all patients with ED [10]. Different studies have shown that testosterone replacement therapy improves metabolic disease and central obesity [11]. 

The objectives of this study were to analyse the relationship between ED, MS, and systemic inflammation. The end point of this case-control study was to investigate the prevalence of metabolic syndrome in patients with ED in comparison with control subjects and to analyse the association with acute phase reactants (CPR, ESR, fibrinogen, D-dimer) and hormone levels. 

## 2. Material and Methods

### 2.1. Patients and Controls

This case-control study included 65 outpatient males, 37 with erectile dysfunction consecutively selected, according to the International Index of Erectile Function (IIEF) from the Urology Department of San Cecilio University Hospital, Granada (Spain) and 28 healthy volunteers controls without erectile dysfunction from outside of the hospital. Participants from both groups answered the IIEF test to determine the presence (or not) of ED. The IIEF is a test with 15 questions about sexual activity, sexual desire, and sexual potency.

Inclusion criteria were male patients, age between 40 and 65 years with erectile dysfunction as defined by the IIEF and signing of the informed consent for study participation. Exclusion criteria were treatment based on an antiandrogen hormone therapy or drugs that cause iatrogenic erectile dysfunction (beta-blocking agents, antidepressants, antipsychotic drugs, etc.). Inclusion criteria for controls were age between 40 and 65 years and signing of the informed consent for study participation. Exclusion criteria for controls were the same as described above and the presence of ED.

### 2.2. Clinical and Laboratory Parameters

The severity of ED was determined by application of the IIEF test. The weight, height, and abdominal circumference of the subjects were measured, and their body mass index (BMI, kg/m^2^) was calculated. Systolic and diastolic blood pressure (BP) was measured after a 5 min rest and again 10 min later; the mean value was recorded.

Serum triglycerides (mg/dL), HDL-C (mg/dL), LDL-C (mg/dL), total cholesterol (mg/dL), glycaemia (mg/dL), insulin (uUI/mL), glycated haemoglobin (%) albumin (mg/dL), SHBG (nmol/L), total testosterone (ng/mL), free testosterone (ng/mL), bioavailable testosterone (ng/mL), FSH (mUI/mL), LH (mUI/mL), prolactin (ng/mL), progesterone (ng/mL), oestradiol (pg/mL), D-dimer (mg/L), fibrinogen (mg/dL), erythrocyte sedimentation rate (ESR, mm/h), and C-reactive protein (CRP, mg/dL) were studied in samples drawn between 8 and 9 a.m. after a rest period of ≥30 min. The homeostasis model assessment of insulin resistance (HOMA-IR index, *μ*U/mg) was also calculated (fasting insulin × fasting glucose/22.5).

Data were also gathered on age, alcohol consumption (>40 g/day), smoking (>5 cigarettes/day), sedentarism (physical exercise <30 min/day), and drug intake (antihypertensives, diuretics, hypocholesterolemics or oral antidiabetics). The prevalence of metabolic syndrome was calculated according to ATP-III criteria. MS was defined by the presence of three of the following: abdominal circumference >102 cm; hypertriglyceridaemia >150 mg/dL, HDL-cholesterol <40 mg/dL, blood pressure >130/85 mmHg, or glycaemia >110 mg/dL. 

Fasting blood samples were obtained between 8 and 9 a.m. after a rest period of ≥30 min by venipuncture of the patients' large antecubital veins without stasis and after a 12-hour fasting period. 

### 2.3. Statistical Analysis

Unpaired, two-sided Student's *t* tests were applied to compare the mean values of quantitative variables, the Kolmogorov-Smirnov test to examine the normality of their distribution, and the Levene test to study the variance. The Mann-Whitney *U* test was used if the variables were not normally distributed. Qualitative variables were analysed with chi-square test or Fisher's exact test if at least one cell had an expected count <5. Correlations among variables were studied by using the Pearson coefficient or Spearman's methods if the variables were nonnormally distributed. Binary logistic regression models (the Wald method) were used to obtain estimated adjusted ORs and their 95% confidence intervals (CIs) to measure the association between ED and metabolic syndrome criteria. Multiple linear regression analysis was used to determine the independent predictors of IIEF, and the standardized coefficient of determination was calculated. Differences were considered significant at *P* ≤ 0.05. The SPSS 17.0 programme was used for the data analyses (SPSS, Inc., Chicago, IL, USA).

### 2.4. Ethics

The study was approved by the Ethics Committee of San Cecilio University Hospital, and written informed consent was obtained from all patients and controls according to the Helsinki Declaration.

## 3. Results

The mean age of patients with ED was 55.8 ± 7.7 years versus 52.5 ± 4.8 years in the control group (*P* = 0.06). The mean BMI in the patient group was 29.8 ± 4.4 kg/m^2^ versus 26.9 ± 4.6 kg/m^2^ in the control group (*P* = 0.01). The IIEF score in patients was 9.7 ± 7.2 versus 29.7 ± 0.5 in the controls (*P* = 0.0001). No significant differences were found between groups in terms of alcohol consumption (48.6% versus 64.3% for patients and controls, resp., *P* = 0.30) or smoking (29.7% versus 42.8% for patients and controls, resp., *P* = 0.25). However, 62.6% of patients admitted that they did not do any exercise versus 28.5% of controls (*P* = 0.006). A total of 29.7% of the patients with ED were previously diagnosed with arterial hypertension versus 25% in the control group (*P* = 0.77), and 32.4% of the patients presented with diabetes mellitus versus 10.7% of the controls (*P* = 0.031). 

### 3.1. Metabolic Syndrome

ATP-III criteria for MS were met by 64.9% of the patients with ED versus 9.5% of the controls (*P* < 0.0001, OR = 17.53, 95% CI: 3.52–87.37). Significant differences in MS parameters between ED patients and controls are listed in Table 1. In the group with ED, the abdominal circumference was 106.6 ± 11.2 cm versus 94.2 ± 11.3 cm in the control group (*P* < 0.0001). Mean systolic and diastolic arterial blood pressure was 151 ± 22 mmHg and 88 ± 10 mmHg, respectively, in the patients and 128 ± 13 mmHg and 78 ± 7 mmHg in the controls (*P* < 0.0001). Median glucose levels were 108 mg/dL in the group with ED versus 93 mg/dL in controls (*P* = 0.005), and median glycated haemoglobin was 5.8% versus 5.6% (*P* = 0.02) for patients and controls, respectively. The MS criteria most frequently recorded in patients with ED were abdominal obesity and systolic and diastolic hypertension. A negative significant correlation between IIEF score and metabolic syndrome parameters was found: systolic BP (*r* = −0.54, *P* = 0.0001), diastolic BP (*r* = −0.43, *P* = 0.0001), abdominal perimeter (*r* = −0.43, *P* = 0.0001), and glucose levels (Spearman's coefficient = −0.45, *P* = 0.0001). Multivariate studies with binary logistic regression showed a strong association between patients with ED and MS even after additional adjustment for age, BMI, IIEF, tobacco use, sedentarism, and alcohol consumption (OR = 20.05, 95% CI: 1.24–32.82, *P* < 0.034, Table 2).

### 3.2. Hormone Study

No significant differences in hormone levels were identified between patients and controls (Table 3). Patients were classified according to total testosterone levels to define the presence of hypogonadism (<3.5 ng/mL). Hypogonadism was found in 21.6% of patients with ED and in 8% of the control group. Patients with hypogonadism did not present with a significantly higher prevalence of ED (*P* = 0.1); however, patients with hypogonadism presented with a significantly higher prevalence of metabolic syndrome (77% of the patients with hypogonadism presented with metabolic syndrome versus 38.8% of the patients without hypogonadism, *P* = 0.031, OR = 2.75, 95% CI = 1.95–9.54). Negative significant correlations were observed between testosterone levels and weight (*r* = −0.29, *P* = 0.03) and triglyceride levels (*r* = −0.27, *P* = 0.04). A positive significant correlation was observed between testosterone levels and HDL-C (*r* = 0.29, *P* = 0.03). Negative significant correlations between SHBG levels and weight (*r* = −0.43, *P* = 0.002), abdominal perimeter (*r* = −0.47, *P* = 0.0001), and BMI (*r* = −0.46, *P* = 0.001) were also found. In addition, there was a significant correlation between insulin levels and glycaemia (Spearman's coefficient = 0.38, *P* = 0.005) and between HOMA-IR and HDL-C (Spearman coefficient's = −0.35, *P* = 0.012).

### 3.3. Acute Phase Reactants

Table 4 shows the mean fibrinogen, D-dimer, ESR and CRP values observed in the study groups. Significant differences in C-reactive protein were found (0.35 ± 0.36 mg/dL versus 0.14 ± 0.11 mg/dL, resp., for patients and controls; *P* = 0.05). No significant differences in median erythrocyte sedimentation rate were found (6 mm/h versus 5 mm/h for patients and controls, resp., *P* = 0.129). Significant correlations between acute phase parameters and IIEF scores or metabolic syndrome criteria are shown in Table 5. Multiple linear regression analysis (Table 6) showed that systolic BP and CRP predicted 0.46 (model *R* ^2^) of IIEF changes (standardised *β* for systolic BP: −0.58, *P* = 0.0001 and standardised *β* for CRP: −0.22, *P* = 0.05).

## 4. Discussion

The results of this study confirm the association between ED and higher cardiovascular risk. We found a higher prevalence of MS (ATP-III criteria) in patients with ED than in control subjects. Binary logistic regression showed a strong association between ED and metabolic syndrome after controlling for confounding variables. In addition, multiple linear regression analysis showed that systolic BP and CRP predicted IIEF changes in a high rate. No significant differences in hormone levels were identified between patients and controls, but patients with hypogonadism presented a higher prevalence of MS.

Diabetes mellitus, arterial hypertension, dyslipidaemia, obesity, and tobacco use are well known risk factors for cardiovascular disease because they are associated with endothelial dysfunction and the development of premature atherosclerosis. This endothelial dysfunction produces ED, due to reduced nitric oxide production and reduced vascular distensibility which inhibits an adequate erection during sexual intercourse [4, 12, 13]. ED has been linked in various studies with CD and MS [5, 6, 13].

In our study, patients with ED have a much higher prevalence of MS than the control group. These results are consistent with other results found in the literature which have shown that MS is strongly associated with ED [14–16]. We have observed that the MS criteria most frequently recorded in patients with ED were abdominal obesity and systolic and diastolic hypertension. These results are similar to those published by Bal et al., who reported that higher blood pressure, fasting glucose, and abdominal perimeter were the risk factors that best predicted the onset of ED [15]. Patients with ED and MS have a reduced elasticity in large arteries, resulting in a higher flow pressure and more arterial hypertension. Patients with ED and MS show a higher risk of cardiovascular events [17]. 

Some biochemical mediators have been measured in patients with suspected endothelial dysfunction [6, 9, 18]. In our study, we have observed that patients with ED had higher levels of C-reactive protein but not higher levels of fibrinogen and D-dimer or a higher erythrocyte sedimentation rate. A statistically significant negative linear correlation between IIEF score and C-reactive protein levels was found, which means that high levels of this mediator are related to the severity of ED. Elevated C-reactive protein levels in patients with ED have been found in patients with endothelial dysfunction [8].

In some patients with ED, the risk for CD and MS is associated with a deficit in testosterone levels [19, 20]. In our study, no significant differences were found in total, free, or bioavailable testosterone levels in patients with ED or the controls. Patients were divided into two groups according to their levels of total testosterone (lower than 3.5 ng/mL and higher to 3.5 ng/mL), and the prevalence of MS and ED in these two groups was analysed. Patients with hypogonadism did not present with higher rates of ED, but they did demonstrate a higher prevalence of MS. These results support the idea that MS may be associated with a deficiency of testosterone levels, and hormone replacement therapy may be useful in these patients [11, 21]. In addition, lower levels of testosterone are associated with higher weight and triglyceride levels and lower HDL-cholesterol, and SHBG correlated negatively with weight, body mass index and abdominal circumference [22–25]. We failed to demonstrate an association between testosterone levels and ED, thus we believe that testosterone does not play an important role in patients with ED when MS is present [10, 26–28]. We also believe that vascular endothelial changes are a common cause of ED [1].

In conclusion, the results obtained show that patients with ED have a higher prevalence of MS and higher mean values of acute phase parameters (CRP). Binary logistic regression analysis showed a strong association between ED and metabolic syndrome after controlling for multiple confounding variables. In addition, multiple linear regression analysis showed that systolic BP and CRP predicted a high percentage of IIEF changes. Cardiovascular screening by MS criteria assessment in patients with ED may be useful to detect individuals who are at risk and start preventive treatment against the development of cardiovascular disease.

## Figures and Tables

**Table 1:** Analysis of the ATP-III metabolic syndrome criteria (mean ± SD when normally distributed, median when nonnormally distributed) in patients with ED and their respectively controls.

	Patients with ED	Controls	*P* value
Systolic blood pressure (mmHg)	151.03 ± 22.16	128.8 ± 13.29	0.0001
Diastolic blood pressure (mmHg)	88.49 ± 10.41	78.88 ± 7.85	0.0001
Abdominal perimeter (cm)	106.62 ± 11.23	94.28 ± 11.33	0.0001
Fasting glucose (mg/dL)	108	93	0.005*
HDL-cholesterol (mg/dL)	47.27 ± 9.45	51.15 ± 11.52	0.177
Triglycerides (mg/dL)	146.59 ± 72.42	155.10 ± 91.68	0.947

*Mann-Whitney *U* test was used for nonnormal variables.

**Table 2:** Binary logistic regression model for metabolic syndrome (dependent variable).

Variable	OR	95% CI	*P* value
Erectile dysfunction (versus control)	20.05	1.24–32.82	0.034
BMI (per unit kg/m^2^)	1.83	0.68–2.43	0.77
Age (per year)	1.07	0.97–1.17	0.16
Alcohol (per gr)	2.65	0.59–11.76	0.20
Smoking (versus no smoking)	2.03	0.37–11.93	0.40
Sedentarism (versus no exercise)	1.31	0.47–3.63	0.60
IIEF (per unit)	1.04	0.93–1.12	0.99

The presence of ED was an independent factor associated with MS.

**Table 3:** Hormonal parameters in patients with ED and controls. No significant differences were found (mean ± SD when normally distributed, median when nonnormally distributed).

Hormonal parameters	Patients with ED	Controls	*P* value
Albumin (g/dL)	4.5 ± 0.2	4.6 ± 0.2	0.12
SHBG (nmol/L)	38.5 ± 19.5	46.7 ± 28.7	0.24
Total testosterone (ng/mL)	4.9 ± 2.2	5.2 ± 1.9	0.71
Free testosterone (ng/mL)	0.09	0.08	0.70*
Bioavailable testosterone (ng/mL)	2.3 ± 0.8	2.2 ± 0.6	0.75
FSH (mUI/mL)	6.1 ± 3.6	4.9 ± 1.7	0.23
LH (mUI/mL)	4.9 ± 2.7	3.9 ± 1.3	0.07
Prolactin (ng/mL)	17.7 ± 4.2	10.7 ± 3.7	0.60
Progesterona (ng/mL)	0.4 ± 0.2	0.5 ± 0.2	0.43
Estradiol (pg/mL)	32.2 ± 12.4	27.6 ± 13.8	0.22
TSH (*μ*UI/mL)	2.7 ± 1.7	2.1 ± 0.9	0.09

^∗^Mann-Whitney *U* test was used for nonnormal variables.

**Table 4:** Mean (SD) of CRP, fibrinogen, D-dimer, ESR, insulin, HOMA-IR, and glycated hemoglobin in patients with ED and their respectively controls (mean ± SD when normally distributed, median when nonnormally distributed).

	Patients with ED	Control group	*P* value
C Reactive protein (mg/dL)	0.35 ± 0.36	0.14 ± 0.11	0.05
Fibrinogen (mg/dL)	299.9 ± 71.9	275.9 ± 75.3	0.23
D dimer (mg/L)	0.29	0.26	0.86*
Erythrocyte sedimentation rate (mm/h)	6	5	0.14*
Insulin (uUI/mL)	16.5 ± 19.9	11.3 ± 13.4	0.33
HOMA-IR	6.04	2.68	0.008*
Glycated hemoglobin (%)	5.8	5.6	0.029*

^∗^Mann-Whitney *U* test was used for nonnormal variables.

**Table 5:** Correlation between acute phase parameters and IIEF score or metabolic syndrome criteria.

Parameters correlation	*r* Pearson or rho Spearman*	*P* value
IIEF-CRP	−0.367	0.007
CRP-sBP	0.308	0.02
CRP-dBP	0.319	0.02
CRP-weight	0.358	0.008
CRP-abdominal perimeter	0.413	0.002
CRP-ESR	0.282	0.04*
CRP-fibrinogen	0.435	0.002
CRP-BMI	0.448	0.001
ESR-fibrinogen	0.314	0.02*

IIEF: International Index Erectile Function; sBP: systolic Blood Pressure; dBP: diastolic Blood Pressure; CRP: C-Reactive Protein; ESR: Erythrocyte Sedimentation Rate.

**Table 6:** Multiple linear regression analysis of independent predictors of IIEF (model adjusted *R* ^2^ = 0.46; *P* = 0.0001).

Predictors	Standardized *β*	*t* value	*P* value
Systolic BP	−0.58	−4.79	0.0001
CRP	−0.22	−1.94	0.048
Insulin	−0.20	−1.82	0.075
Age	0.10	0.86	0.39
Constant	52.3	4.83	0.0001
